# Technological interventions for loneliness and social isolation among older adults: a scoping review protocol

**DOI:** 10.1186/s13643-021-01775-6

**Published:** 2021-08-07

**Authors:** Andrew Wister, Ian Fyffe, Eireann O’Dea

**Affiliations:** Gerontology Research Center, Simon Fraser University, 2800-515 West Hastings St, Vancouver, V6B 5K3 Canada

**Keywords:** Older adults, Social isolation, Loneliness, Technology, Intervention, Program, Physical

## Abstract

**Background:**

Loneliness and social isolation are prevalent public health concerns among community-dwelling older adults. One approach that is becoming an increasingly popular method of reducing levels of loneliness and social isolation among older adults is through technology-driven solutions. This protocol outlines a research trajectory whereby a scoping review will be initiated in order to illustrate and map the existing technological approaches that have been utilized to diminish levels of loneliness and social isolation among community-dwelling older adults aged 60 years or older. We will address the question: *what are the most common and less used technological approaches to reduce loneliness and social isolation among community-dwelling older adults?*

**Methods:**

A scoping review of Academic Search Premier, AGEline, Global Health, MEDLINE, PsycINFO, and Web of Science databases will take place using our search terms including the following: loneliness, social isolation, older adults, elderly, Aged, Aged 80 and over, program, evaluation, trial, intervention, technology, computer, information and communication technology, internet, and robot. The initial electronic search will be supplemented by reviewing the reference lists and review articles to identify any missing studies. To meet study inclusion criteria, intervention studies had to pertain to community-dwelling adults aged 60 years or older, include technological interventions, include loneliness and/or social isolation as outcome variables, and be written in the English language. Two parallel independent assessments of study eligibility will be conducted for the title, abstract, and full-text screens. Any disagreement will be resolved by consensus and a third reviewer consulted to make a decision if consensus is not achieved initially. Finally, the amalgamation of results will be an iterative process whereby reviewers will refine the plan for presenting results after data extraction is completed so that all of the contents of the extraction may be included in the results.

**Discussion:**

The information gleaned in this scoping review will be essential to understand the degree to which technological interventions influence social isolation and loneliness among older adults and identify gaps for further research.

## Background

Loneliness and social isolation are unique yet interrelated concepts with multiple definitions, conceptualizations, and measurements in the literature. There is agreement that loneliness is the subjective perception that intimate and social needs are not being met. Whereas, social isolation refers to a multifaceted objective condition in which there is an absence of social engagement and social connectedness within family, friendship and community social networks [43]. Between one third and one quarter of older adults will experience loneliness and/or social isolation; and about 10% will experience chronic levels of these conditions [13, 41, 43].

Loneliness and social isolation are prevalent public health concerns among community-dwelling older adults. Loneliness and social isolation are associated with reduced happiness, life satisfaction, and psychological well-being; increased depression; compromised physical and mental health; and higher mortality among older adults [7, 12, 21, 44]. Furthermore, these social-psychological contexts have been associated with lower access to health care services, and lower health care utilization in older age [29]. For these reasons, many community and long-term care organizations that serve older adults have developed outreach programs that aim to reduce levels of loneliness and social isolation through technologically driven solutions [3, 5]. These technologically driven solutions include, but are not limited to, interventions such as internet and communication technology (ICT), telephone, social media (e.g., Facebook), videoconference technology (e.g., Zoom), robotics (e.g., robotic companions), and video gaming (e.g., Nintendo Wii). However, most of these developments have not been evaluated for efficacy of the intervention under ideal conditions, or effectiveness of the intervention in terms of application in natural settings. This is complicated by the fact that it is often difficult to isolate the ‘technology effect’ from the ‘human effect’ for interventions that combine these elements. For example, an ICT intervention that focuses on teaching computer skills may have a classroom element whereby participants socialize with each other, which may influence the study outcome of loneliness. In addition, there is a ‘digital divide’ for some older adults that prevent them from either accessing technology or having the technological literacy to use them effectively [25]. While the program evaluation literature in this area is not developed to the level that allows for in-depth meta-evaluation or meta-analyses, there is opportunity for a scoping review to identify technological approaches to reducing loneliness and social isolation that show promise.

The influencing aspects of loneliness and social isolation on the effectiveness of technological interventions should also be noted. For interventions that involve the accessing of an existing social network, such as videoconferencing or social media, chronic social isolation may interfere with the effectiveness of the technological intervention, thus posing a Catch-22. For instance, individuals with very small social networks limit access to the very resources that the intervention seeks to harness to address levels of loneliness and social isolation. This may include individuals who have been estranged from their families as well as those with chronic mental illnesses, such as those with severe forms of schizophrenia (see [8].

Technological approaches to loneliness and social isolation can be conceptualized using a resilience model of aging. Resilience is defined as the ability to adapt and thrive when faced with one or more adversities [42]. Risk and resilience factors for adversity include a range of known social, social-psychological and epidemiological influences (e.g., social network, stressors, environmental, cultural, lifestyle, behavioral, and demographic factors such as age and gender), some of which are mutable (physical activity), and some of which are not (genetics). This approach is built upon three interlocking models: (1) a complex systems model [18], (2) a socio-ecological model [38], and (3) a social determinants of health model [33]. A resilience model combines analyses of system-level function and links and quantifies the different individual and environmental-level social determinants of health observed within existing socio-ecological frameworks [46]. A resilience model of aging can be used to illustrate how an individual at risk of loneliness and/or social isolation may be socially resilient due to accumulated positive experiences overcoming adversity by accessing social support networks [45]. This could be possible by utilizing technology such a social media and/or videoconferencing technology to access either existing informal social networks or those residing in the community that may otherwise be difficult to reach physically.

Turning to the role of interventions, Verbrugge and Jette ([40]:8) contend that social risk/protective factors of disablement processes entail: effective interventions (e.g., medical care, programs, services, technological aids) and exacerbators of the disablement process among older adults (e.g., poor interventions, social isolation due to fear of falling, and age-unfriendly environments). The latter increase the chances of disease and subsequent disability, but which are modifiable. It is expected that technology plays a critical role in allowing community-dwelling older adults to retain healthy levels of social engagement by providing a substitute for face-to-face social interaction, or a complementary model. For community organizations that target their support systems to less resilient socially isolated older adults, technological innovations may enrich their outreach programs in a number of ways. This embodies diffusion of technological communication devices, their interface, and related training programs, technology donation programs, and other innovations that foster social connectedness in a spectrum of forms. Program strategies may entail transitioning outreach programs from a face-to-face format to an online format, use of low-tech options such as the phone for older adults who have limited access or knowledge, or combining technology-driven and human-driven approaches. Advancement of technological applications to assist older adults that meet a variety of functional and emotional needs has been rapid in recent years, requiring updating of literature reviews, especially during the COVID-19 pandemic, during which time formal and informal systems are being adapted, sustained, and improved [10, 19, 37].

This proposed scoping review provides a unique perspective that is differentiated from recent reviews, including targeted systematic reviews (see [5, 15, 17, 36] in several ways. Our review includes studies exclusively on social isolation and loneliness among community-dwelling older adult populations as opposed to: excluding loneliness [17], including social connectedness [15] and institutionalized populations (e.g., [35]. Our scoping protocol also does not concentrate on sub-populations such as individuals with dementia (e.g., [26]. Additionally, our scoping review focuses on all forms of technology compared to other reviews that only review distinct sub-types of technology such as social networking sites or only digital technology (e.g., [4, 36], information sites [5], video calls , or robots (e.g., [1]. For these reasons, we believe that a scoping review of this kind would be beneficial to the literature at this time.

## Methods

### Study objectives

The objective of this scoping review is to illustrate and map the existing technological approaches that have been utilized to diminish levels of loneliness and social isolation among community-dwelling older adults aged 60 years or older. We intend on addressing the research question: *what are the most common and less known technological approaches to reduce loneliness and social isolation among community-dwelling older adults?* This will be achieved by identifying and mapping the concepts used in published works, and evaluating the types and quality of data from each source in a format that follows from our study objective [2, 27]. The mapping of these data will inform our next study, a comprehensive systematic review of randomized controlled trial studies of technological interventions on loneliness and social isolation. The results of the systematic review study will be shared with local, provincial and national stakeholders who currently operate not-for-profit loneliness intervention programs for seniors within the broader community of Greater Vancouver, Canada.

### Study design

The scoping review methodology outlined by the Joanna Briggs Institute’s Reviewer Manual [32] will be followed in order to meet the study objective. This methodological standard was originally published in 2015 to address problems in the scoping review literature. Up until that point, it was noticed that the scoping review literature lacked consistency in both terminology and methods reported [6]. We propose to begin with a scoping review in this field, followed by a systematic review. A scoping review methodology has been chosen over a systematic review methodology, since the former has a purpose that is consistent with the development of literature in this field [39]. Systematic reviews are intended for uncovering international evidence, confirming current practices or identifying new practices, identifying and informing areas for future research, identifying and investigating conflicting results, and/or producing statements to guide decision-making [28]. Although systematic review articles on technology and social isolation/loneliness have been published, these have been limited to studies in focused sub-areas of technology and/or sub-populations (e.g., communication technologies, robotics, dementia patients) (see [3, 5, 16]) and not on community-based older adults more broadly.

Conversely, the purposes of scoping reviews are to map the key concepts that underlie a field of research, clarify working definitions, and/or clarify the conceptual boundaries of a subject, and generate hypotheses for research [2, 39]. In line with a scoping review, the objective of this review is to identify and map the types of available evidence in this rapidly evolving field [27]. Further, this scoping review protocol and the corresponding scoping review article are intended to be precursors to a systematic review article that will update prior systematic reviews and will capture the upsurge in this area during COVID-19.

The scoping review will be performed by a team of three researchers — a professor, doctoral university student, and a research assistant. The academic professor will serve as a guide to provide overall direction and support for the study. The review will be performed by the other two members through five stages outlined by Hung et al. [14]: conducting broad searches, refining selection criteria, study selection and reviewing results, mapping literature, and summarizing results. The JBI endorsed Preferred Reporting Items for Systematic Reviews and Meta-Analyses for Scoping Reviews (PRISMA-ScR) checklist will also be applied in order to structure the reporting of the scoping review. Prior to screening, reviewers will undergo training to ensure basic understanding of the background of the field and purpose of the review.

### Eligibility criteria

The stages of this scoping review will follow Arksey and O’Malley [2] proposed guidelines. The JBI utilizes the PCC (population, concept, and context) mnemonic to guide the research question(s) and inclusion criteria (see Table 1). For population, we will consider only studies with populations of 60 years of age or older. In the case of studies where only a mean age is reported, then a mean age of 65 will be sufficient. For concept, we will include studies of technological interventions on the outcomes of loneliness and/or social isolation. Types of technology interventions expected for this review include education on the use of technology, interventions utilizing robot technology, social networking-based interventions, and videoconferencing. In terms of context, we will include studies of community-dwelling older adults only. Studies that include populations of both older adults living in the community and in residential facilities will be included, as long as outcomes are separated by living arrangement. Studies must be published in the English language. Technological interventions on institutionalized populations and adults aged younger than 60 years will be excluded. In accordance with the JBI, an assessment of methodological quality will not be performed in this review [32] but will be incorporated into the subsequent systematic review. The range of evidence will include different methodologies from qualitative to quantitative in nature. Sources as gray literature and unpublished theses and dissertations will be included in this review in order to maximize the number of potential studies for inclusion.

**Table 1:** The PCC mnemonic and inclusion criteria

PCC	Inclusion criteria
Population	• Older adults (aged 60 or older)
Concept	• Intervention (technological) • Outcomes (loneliness, social isolation)
Context	• Community

### Information sources

The initial search terms will be applied in six databases: Academic Search Premier, Ageline, Global Health, MEDLINE, PsycINFO, and Web of Science. Additional sources will be found by searching the reference lists of articles. Review articles found in the database search stage will also be utilized for reference harvesting. These additional measures will ensure that the sources that were discovered in the original search will be bolstered.

### Search strategy

The database coverage will be any date to August 2020, and the English language will be applied as a limit to remain consistent with eligibility criteria. The following keywords will be used: loneliness, social isolation, older adults, elderly, seniors, geriatrics, program, evaluation, trial, intervention, technology, computer, information and communication technology, internet, and robot. Various subject headings (i.e., MeSH) will also be utilized depending on the database being searched. An example of a search strategy for the database MEDLINE (EBSCO) is as follows:

Search: ( MM Aged [MeSH term] OR MM “Aged, 80 and over”[MeSH term] OR TX “older adults” OR TX elderly ) AND ( MM technology [MeSH term] or technology [All Fields] OR MM “User-Computer Interface” OR TX Computers OR MM internet [MeSH term] OR internet [All Fields] OR MM “computers, handheld” [MeSH term] OR MM “Information and communication technology” [MeSH term] OR Robot [All Fields] ) AND ( MM loneliness [MeSH term] OR loneliness [All Fields] OR MM “social isolation” [MeSH term] OR social isolation [All Fields] ) AND ( intervention [All Fields] OR program [All Fields] OR evaluation [All Fields] OR trial [All Fields])

The proposed line-by-line search strategies for each database is included in Appendix.

### Study records

#### Data management

In order to expedite the construction of a bibliography, Zotero — a bibliographic reference management tool — will be used. Zotero will be employed in order to arrange and structure all articles. Sources found during the three stages presented above will be uploaded into Zotero and duplicates will be removed. The data analysis software NVivo 12 will be utilized to extract data from selected articles during the full-text review process. This extracted data from the articles will be inputted into the data extraction tool.

#### Selection process

In order to determine whether the independent researchers are utilizing an approach that is consistent with the purpose and research question, the first twenty studies will be independently reviewed and discussed by the doctoral student and the research assistant. This strategy is consistent with that which has been articulated by Levac et al. [22]. This step will ensure early calibration and correction of any systematic patterns of discrepancies that may arise between reviewers and to determine whether the instructions for screening are sufficient.

The review process will include two stages of screening performed by two independent reviewers. The first screening stage is a title and abstract review for appropriateness. Inclusion and exclusion criteria will be applied to the selected articles at this stage. If the relevance of an abstract is unclear, full-text screening will be conducted [2]. The second screening stage is a full-text review for inclusion or exclusion. Reasons for exclusion will be noted and presented in a PRISMA flow diagram. Disagreements between reviewers regarding the inclusion or exclusion of articles will be resolved via discussion. If necessary, the professor will advise the reviewers in an instance where a disagreement cannot be resolved among the reviewers themselves. This situation will be resolved by the whole team, however, and not in a top-down fashion***.***

#### Data collection process

A data extraction tool based on the inclusion criteria will be created with the input from the entire team and will be generated by using the JBI Reviewer’s Manual Appendix 11.1 [32] as a template. The data extraction tool will be utilized by two independent reviewers. Due to the comprehensive nature of the data extraction tool, it is not expected that alterations will be required. However, any potential alterations to the data extraction tool will be detailed extensively in the full scoping review.

### Data items

We will extract the type of technological intervention utilized to reduce loneliness and/or loneliness among older adults, the type of instrument used to measure loneliness and/or social isolation, study design, n-size, and the age range or mean age of participants.

### Outcomes

Outcomes of interest in this study include loneliness and social isolation. Studies reviewed may include multiple definitions for these terms; however, definitions should be similar to those outlined earlier in this protocol, with loneliness defined as the subjective perception that intimate and social needs are not being met, and social isolation referring to a multifaceted objective condition in which there is an absence of social engagement and social connectedness within family, friendship, and community social networks [43]. Examples of expected measures for loneliness and social isolation include the De Jong Gierveld Loneliness Scale (De Jong [11], the Duke social support index-10 [20], and the UCLA Loneliness Scale [34].

#### Visual presentation: flow diagram and mapping literature

The scoping review process will be presented visually in two ways: in a PRISMA flow diagram and in a literature map that charts the data. The scoping review process will be detailed in a flow diagram based upon the one adapted from the PRISMA statement by Moher et al. [24]. This promotes greater transparency into the review and helps allow for replication. In order to best summarize the results, the selected articles will be mapped in a logical formation. The review findings will be presented based upon suggested JBI categories such as year of publication, countries of origin, and research methods [32]. This information will be derived and summarized from the comprehensive data extraction tool.

#### Summarizing results

The findings of this review will be reported in a peer-reviewed academic journal. Aside from the PRISMA flow diagram and the literature map, the results will be summarized throughout the manuscript. The purpose of this narrative summary will be to describe how the results pertain to the review objective.

### Ethics and dissemination

Ethics approval is not required at our institution for this study, since the methodology involves collecting data from secondary sources. The methodology outlined by this scoping review protocol is a transparent one that will produce an ethical research project. That scoping review will be published in a peer-reviewed academic journal. Furthermore, the findings from that project will be utilized in a subsequent research project: a systematic review focusing on quantitative RCT technological interventions for loneliness and social isolation.

## Discussion

The results of the scoping study will generate a comprehensive list of various technologies that have been utilized to diminish levels of loneliness and social isolation among community-dwelling adults aged 60 years or older. While there exists several scoping/systematic reviews in the literature in relation to this topic [23]; they are either extremely broad (e.g., [9, 31] or are concentrated on one specific form of technology, such as robotics [1] or internet-based digital tools [30]. Our approach differs in that all possible technological interventions for loneliness and social isolation are considered for community-based older adults. This allows for our scoping review to be used as a foundation for a subsequent systematic review and eventually meta-evaluation research that compares the advantages and disadvantages of different kinds of technological interventions.

## Data Availability

Data sharing is not applicable to this article as no datasets were generated or analyzed during the current study.

## Appendix

Search strategy

PscyhINFO (EBSCO)

Search: (Aging [All fields] OR AB “older adults” or AB elderly) AND (AB technology OR Computers [All Fields] OR internet [All Fields] OR “information and communication technology” [All fields] OR “robot” [All Fields] ) AND ( AB loneliness OR loneliness [All fields] OR AB “social isolation” OR “social isolation” [All Fields] ) AND ( AB intervention OR program [All Fields] OR evaluation [All Fields] OR trial [All Fields] )

Anytime-2020, English

= 115 results

AgeLine (EBSCO)

Search: (TX technology OR Computers [All Fields] OR internet [All Fields] OR “information and communication technology” [All Fields] OR “robot” [All Fields]) AND (TX loneliness OR TX “social isolation”) AND (intervention [All Fields] OR program [All Fields] OR evaluation [All Fields] OR trial [All Fields] )Anytime- 2020, English

= 76 results

Academic Search Premier (EBSCO)

Search: (“older people” [All Fields] OR “older adults” [All Fields] OR elderly [All Fields]) AND (technology [All Fields] OR computers [All Fields] OR internet [All Fields] OR “information and communication technology” [All Fields] OR robot [All Fields]) AND (loneliness [All Fields] OR “social isolation” [All Fields] ) AND (intervention [All Fields] or program [All Fields] or evaluation [All Fields] or trial [All Fields])Anytime- 2020, English

= 198 results

MEDLINE (EBSCO)

Search: ( MM Aged [MeSH term] OR MM “Aged, 80 and over”[MeSH term] OR TX “older adults” OR TX elderly ) AND ( MM technology [MeSH term] or technology [All Fields] OR MM “User-Computer Interface” OR TX Computers OR MM internet [MeSH term] OR internet [All Fields] OR MM “computers, handheld” [MeSH term] OR MM “Information and communication technology” [MeSH term] OR Robot [All Fields] ) AND ( MM loneliness [MeSH term] OR loneliness [All Fields] OR MM “social isolation” [MeSH term] OR social isolation [All Fields] ) AND ( intervention [All Fields] OR program [All Fields] OR evaluation [All Fields] OR trial [All Fields])Anytime-2020, English

= 281 results

Global Health

Search: (“older adults” [All Fields] OR elderly [All Fields]) AND (technology [All Fields] OR computers [All Fields] OR internet [All Fields] OR “information and communication technology” [All Fields] OR robot [All Fields]) AND (loneliness [All Fields] or “social isolation” [All Fields]) AND (intervention [All Fields] or program [All Fields] or evaluation [All Fields] or trial [All Fields]): Anytime-2020, English

= 11 results

Web of Science

Search: (“older adults” [All Fields] OR elderly [All Fields]) AND (technology [All Fields] OR computers [All Fields] OR internet [All Fields] OR “information and communication technology” [All Fields] OR robot [All Fields]) AND (loneliness [All Fields] OR “social isolation” [All Fields]) AND (intervention [All Fields] OR program [All Fields] OR evaluation [All Fields] OR trial [All Fields]): Anytime- 2020, English

= 335 results

## Abbreviations

COVID-19: Novel coronavirus disease
ICT: Information and communications technology
JBI: Joanna Briggs Institute
PCC: Population, concept, and context
PRISMA-ScR: Preferred Reporting Items for Systematic Reviews and Meta-Analyses for Scoping Reviews
RCT: Randomized controlled trial

## References

[1] Abdi J, Al-Hindawi A, Ng T, Vizcaychipi MP. Scoping review on the use of socially assistive robot technology in elderly care. BMJ Open. 2018;8. 10.1136/bmjopen-2017-018815. 10.1136/bmjopen-2017-018815 PMC5829664 29440212

[2] Arksey H, O’Malley L (2005). Scoping studies: towards a methodological framework. Int J Soc Res Methodol.

[3] Baker S (2018). Combatting social isolation and increasing social participation of older adults through the use of technology: a systematic review of existing evidence. Australas J Ageing.

[4] Casanova G, Zaccaria D, Rolandi E, Guaita A. The effect of information and communication technology and social networking site use on older people’s well-being in relation to loneliness: review of experimental studies. J Med Internet Res. 2021;23(3):e23588. 10.2196/23588 PMC7961406 33439127

[5] Chen, Y.R.R. & Schulz, P.J. (2016). The effect of information communication technology interventions on reducing social isolation in the elderly: a systematic review. Journal of Medical Internet Research, 18(1). DOI: 10.2196/jmir.4596 10.2196/jmir.4596 PMC4751336 26822073

[6] Colquhoun HL (2014). Scoping reviews: time for clarity in definition, methods, and reporting. J Clin Epidemiol.

[7] Courtin E, Knapp M (2015). Social isolation, loneliness and health in old age: a scoping review. Health Soc Care Community.

[8] Degnan, A., Berry, K., Sweet, D., Abel, K., Crossley, N., & Edge, D. Social networks and symptomatic and functional outcomes in schizophrenia: a systematic review and meta-analysis. Soc Psychiatry Psychiatric Epidemiol. 2018; 53(9):873–888.Fakoya, O.A., 10.1007/s00127-018-1552-8 PMC6133157 29951929

[9] Fakoya OA, McCorry NK, Donnelly M (2020). Loneliness and social isolation interventions for older adults: a scoping review of reviews. BMC Public Health.

[10] Gardner W, States D, Bagley N (2020). The coronavirus and the risks to the elderly in long-term care. J Aging Soc Policy.

[11] Gierveld JDJ, Tilburg TV (2006). A 6-item scale for overall, emotional, and social loneliness: Confirmatory tests on survey data. Res Aging.

[12] Golden J (2009). Loneliness, social support networks, mood and wellbeing in community-dwelling elderly. Int J Geriatr Psychiatry.

[13] Grenade L, Boldy D (2008). Social isolation and loneliness among older people: Issues and future challenges in community and residential settings. Aust Health Rev.

[14] Hung L. et al. Use of touch screen tablets to support social connections and reduce responsive behaviours among people with dementia in care settings: a scoping review protocol. BMJ Open. 2019:9. 10.1136/bmjopen-2019-031653 10.1136/bmjopen-2019-031653 PMC6886971 31748304

[15] Ibarra F, Baez M, Cernuzzi L, Casati F. A systematic review on technology-supported interventions to improve old-age social wellbeing: loneliness, social isolation, and connectedness. J Healthcare Eng. 202010.1144/2020/2036842 10.1155/2020/2036842 PMC7374226 32765823

[16] Khosravi P, Ghapanchi AH (2016). Investigating the effectiveness of technologies applied to assist seniors: a systematic literature review. Int J Med Informatics.

[17] Khosravi P, Rezvani A, Wiewiora A (2016). The impact of technology on older adults’ social isolation. Comput Hum Behav.

[18] Klasa K, Galaitsi S, Trump B, Linkov I. Science and practice of resilience: disaster systems applications to aging model development. In A. Wister & T. Cosco (Eds.). Resilience and Aging: Emerging Science and Future Possibilities. New York: Springer. 2020 pp. 53-80.

[19] Luchetti, M., et al. The trajectory of loneliness in response to COVID-19. American Psychologist. 2020. Advance online publication 10.1037/amp0000690. 10.1037/amp0000690 PMC7890217 32567879

[20] Landerman R, George LK, Campbell RT, Blazer DG (1998). Alternative models of the stress buffering hypothesis. Am J Community Psychol.

[21] Leigh-Hunt N, Bagguley D, Turner V, Turnbull N, Valtorta N, Caan W (2017). An overview of systematic reviews on the public health consequences of social isolation and loneliness. Public Health.

[22] Levac D, Colquhoun H, O’Brien KK (2010). Scoping studies: advancing the methodology. Implement Sci.

[23] McCorry NK, Donnelly M (2020). Loneliness and social isolation interventions for older adults: a scoping review of reviews. BMC Public Health.

[24] Moher, D., Liberati, A., Tetzlaff, A., & Altman, D.G. (2009). Preferred reporting items for systematic reviews and meta-analysis: the PRISMA statement. PLoS Med. 6(7). DOI: 10.1371/journal.pmed.1000097. 10.1371/journal.pmed.1000097 PMC2707599 19621072

[25] Morrow-Howell, N., Galucia, N., & Swinford, E. Recovering from the COVID-19 pandemic: a focus on older adults. J Aging Soc Policy. 2020;1. 10.1080/08959420.2020.1759758 32336225

[26] Moyle, W., Jones, C. J., Murfield, J. E., Thalib, L., Beattie, E. R., Shum, D. K., ... & Draper, B. M. Use of a robotic seal as a therapeutic tool to improve dementia symptoms: a cluster-randomized controlled trial. J Am Med Directors Assoc. 2017;18(9), 766-773. 10.1016/j.jamda.2017.03.018 28780395

[27] Munn, Z., et al. Systematic review or scoping review? Guidance for authors when choosing between a systematic or scoping review approach. BMC Med Res Methodol. 2018a;18(1). 10.1186/s12874-018-0611-x PMC6245623 30453902

[28] Munn, Z., et al. What kind of systematic review should I conduct? A proposed typology and guidance for systematic reviewers in the medical and health sciences. BMC Medical Research Methodol. 2018b;18(1), 5-14. 10.1186/s12874-017-0468-4 PMC5761190 29316881

[29] Newall N, McArthur J, Menec VH (2015). A longitudinal examination of social participation, loneliness, and use of physician and hospital services. J Aging Health.

[30] Newman, K., Wang, A.H., Wang, A.Z.Y., & Hanna, D. The role of internet-based digital tools in reducing social isolation and addressing support needs among informal caregivers: a scoping review. BMC Public Health. 2019;19.10.1186/s12889-019-7837-3 10.1186/s12889-019-7837-3 PMC6842183 31706294

[31] O’Rourke HM, Collins L, Sidani S (2018). Interventions to address social connectedness and loneliness for older adults: a scoping review. BMC Geriatr.

[32] Peters, M.D.J., et al. (2020). Chapter 11: scoping reviews (2020 version). In E. Aromataris, Z. Munn, (Eds.). Joanna Briggs Institute Reviewer’s Manual, JBI. Retrieved from https://reviewersmanual.joannabriggs.org/

[33] Rootman I (2012). Health promotion in Canada: critical perspectives on practice.

[34] Russell D (1996). UCLA Loneliness Scale (Version 3): Reliability, validity, and factor structure. J Pers Assess.

[35] Sarabia M, Young N, Canavan K, Edginton T, Demiris Y, Vizcaychipi MP (2018). Assistive robotic technology to combat social isolation in acute hospital settings. Int J Soc Robot.

[36] Shah SGS, Nogueras D, van Woerden HC, Kiparoglou V (2020). Are digital technology interventions effective to reduce loneliness in older adults?. A systematic review and meta-analysis medRxiv.

[37] Shahid Z (2020). COVID-19 and older adults: what we know. J Am Geriatr Soc.

[38] Stokols D (1992). Establishing and maintaining healthy environments: toward a social ecology of health promotion. Am Psychol.

[39] Tricco AC, et al. A scoping review on the conduct and reporting of scoping reviews. BMC Med Res Methodol. 2016:(1):15. 10.1186/s12874-016-0116-4 PMC4746911 26857112

[40] Verbrugge L, Jette A (1994). The disablement process. Soc Sci Med.

[41] Victor CR, Scambler SJ, Bowling ANN (2005). The prevalence of, and risk factors for, loneliness in later life: a survey of older people in great Britain. Ageing Soc.

[42] Wister A, Coatta K, Schuurman N, Lear S, Rosin M, MacKey D (2016). A Lifecourse model of multimorbidity resilience: theoretical and research developments. Int J Aging Hum Dev.

[43] Wister, A., Menec, V., & Mugford, G. Chapter 5: loneliness, social isolation, and social engagement. In P. Raina, C. Wolfson, S. Kirkland, & L. Griffith (Eds.), The Canadian Longitudinal Study on Aging (CLSA) Report on Health and Aging in Canada: Findings from Baseline Data Collection 2010–2015. 2018. Retrieved from http://www.clsa-elcv.ca/doc/2639.

[44] Wister A, Cosco T, Mitchell B, Menec V, Fyffe I (2019). Development and concurrent validity of a composite social isolation index for older adults using the Canadian Longitudinal Study on Aging. Can J Aging.

[45] Wister A, Speechley M (2020). COVID-19: pandemic risk, resilience and possibilities for aging research. Can J Aging.

[46] Wister AV. “Health promotion and aging during a pandemic: risk, resilience and COVID-19.” In I. Rootman, P. Edwards, M. Levasseur & F. Grunberg (Eds). Health of Older Adults: The Canadian Experience. Toronto, ON: Canadian Scholars Press Inc; 2021

